# Novel Predictive Scoring System for Intravenous Immunoglobulin Resistance Helps Timely Intervention in Kawasaki Disease: The Chinese Experience

**DOI:** 10.1155/2023/6808323

**Published:** 2023-08-09

**Authors:** Yan Wang, Shuoyin Huang, Panpan Wang, Yan Wu, Yingying Liu, Yuting Pan, Jinyuan Dong, Zhidan Fan, Haiguo Yu

**Affiliations:** ^1^Department of Rheumatology and Immunology, Children's Hospital of Nanjing Medical University, Nanjing 210008, China; ^2^Department of Child Health Care, Children's Hospital of Nanjing Medical University, Nanjing, China

## Abstract

**Background:**

Approximately 10%–20% of patients with Kawasaki disease (KD) are nonresponsive to intravenous immunoglobulin (IVIG) treatment, placing them at higher risk of developing coronary heart lesions. Early detection of nonresponsiveness is crucial to curtail this risk; however, the applicability of existing predictive scoring systems is limited to the Japanese population. Our study aimed to identify a predictive scoring system for IVIG resistance in KD specific to the Chinese population. We aimed to assess the utility of three commonly used risk-scoring systems in predicting IVIG resistance and compare them to the newly developed predictive scoring system.

**Methods:**

A total of 895 patients with KD were enrolled in this retrospective review and divided into two groups: IVIG responders and nonresponders. Clinical and laboratory variables were compared between the two groups. Multivariable logistic regression models were used to construct a new scoring system. The utility of the existing and new scoring systems was assessed and compared using the area under the receiver operating characteristic curve.

**Results:**

Albumin levels, percentage of neutrophils, and hemoglobin were independent predictors of resistance by logistic regression analysis. The new predictive scoring system was derived with improved sensitivity (60.5%) and specificity (87.8%). The area under the receiver operating characteristic curve was 0.818.

**Conclusion:**

This study developed a novel risk-scoring system for predicting resistance to IVIG treatment in KD specific to the Chinese population. Although this new model requires further validation, it may be useful for improving prognostic outcomes and reducing the risk of complications associated with KD.

## 1. Introduction

Kawasaki disease (KD) is a type of acute febrile systemic vasculitis. While the exact pathogenesis remains unclear, infection, immunity, and genetics are considered possible contributing factors [1]. KD is a common cause of childhood-acquired heart disease worldwide [2, 3]. The most severe sequela of KD is considered to be the development of coronary artery lesions (CALs), which occurs in 20%–25% of untreated patients [4]. At present, the most accepted treatments are intravenous immunoglobulin (IVIG) and aspirin, which can substantially decrease the risk of CAL development. However, in approximately 10%–20% of patients with KD, persistent or recurrent fever occurs despite completion of the initial IVIG administration. Such patients are classified as nonresponsive to IVIG treatment. Mounting evidence indicates that IVIG nonresponders are at higher risk of CALs [5, 6]. Therefore, the early identification of IVIG resistance in patients with KD may play a pivotal role in curtailing this risk.

Several predictive models have been published to predict IVIG resistance in patients with KD such as the Kobayashi scoring system [7], Egami scoring system [8], and Sano scoring system [9]. These scoring systems were developed for the Japanese population, and their applicability in predicting IVIG resistance in other populations are limited.

Therefore, the aim of our study was to identify the new predictors for IVIG resistance in KD and develop a new predictive scoring system specific to the Chinese population.

## 2. Materials and Methods

The clinical records of all patients with KD treated between January 2019 and June 2020 at Nanjing Children's Hospital in China were collected for this retrospective study. This study was approved by the institutional review board of Nanjing Children's Hospital and was conducted in accordance with the Helsinki Declaration of 1964, as revised in 2000. The diagnostic criteria for complete and incomplete KD were based on the Diagnostic Guidelines for KD [10, 11]. Temperature ≥37.5°C was defined as fever. The first day of fever was defined as the first day of the illness. All patients received high-dose IVIG (2 g/kg dose) and oral aspirin (30–50 mg/kg/day) as the initial treatment during the first 10 days of illness. The patients treated with glucocorticoids before or together with initial IVIG therapy, or those treated after 10 days of illness were excluded. Patients diagnosed with KD several months ago and those who visited our hospital for complications such as CALs were also excluded. IVIG nonresponsiveness was defined as persistent or recrudescent fever 48 hr to 2 weeks after initial IVIG treatment, with the presence of at least one of the KD diagnostic criteria [12, 13].

The collected data included sex, age, and laboratory variables before IVIG therapy, including erythrocyte sedimentation rate (ESR), brain natriuretic peptide (BNP), procalcitonin (PCT), serum ferritin (SF), T-lymphocyte count, CD4+ T lymphocyte count, serum soluble interleukin-2 receptor (sIL-2R), interleukin-6 (IL-6), alanine aminotransferase (ALT), aspartate aminotransferase (AST), albumin, total bilirubin, direct bilirubin, sodium, C-reactive protein (CRP), white blood cell (WBC) count, percentage of neutrophils (% neutrophils), hemoglobin (Hb), and platelet (Plt) count. The three risk-scoring systems listed in Table S1 for identifying the IVIG responders and nonresponders in KD were also assessed.

SPSS statistics (IBM. SPSS statistics for Windows. Version 25.0. Armonk, NY: IBM; 2020.) was used for statistical calculations. Continuous values were compared using Student's *t*-test or Mann–Whitney *U* tests, and categorical data were compared using the *χ*^2^ test. *P* < 0.05 was considered to be statistically meaningful. Data were presented as number (%) or mean ± standard deviation. Multivariable logistic regression analysis models were constructed using the variables that were significantly different between the IVIG responders and nonresponders in univariate analysis to predict IVIG nonresponsiveness. A new risk-scoring system was created according to the logistic coefficients of significant predictors. A receiver operating characteristic (ROC) curve was used to evaluate the performance of the predictive models in terms of sensitivity and specificity.

## 3. Results

The study included a total of 895 patients with KD, of which 192 were excluded as follows: 166 patients were treated with glucocorticoids before or together with initial IVIG therapy, 18 patients received late treatment, and 8 patients were diagnosed with KD several months ago and visited our hospital for complications. The remaining 703 patients comprised of 665 (94.6%) IVIG responders and 38 (5.4%) IVIG nonresponders. A comparison of clinical and laboratory variables between IVIG responders and nonresponders is presented in Table 1. Sex was not significantly different between the two groups: the percentage of male IVIG responders was 60.60%, whereas the percentage of male IVIG nonresponders was 68.42%. Further, the average ages of IVIG responders and IVIG nonresponders (26.54 and 32.24 months, respectively) were not significantly different. Comparison of laboratory variables showed that IVIG nonresponders had significantly higher PCT (*P* = 0.036), SF (*P* = 0.003), sIL-2R (*P* = 0.021), ALT (*P* = 0.030), total bilirubin (*P* = 0.004), direct bilirubin (*P* = 0.004), CRP (*P* = 0.002), WBC count (*p* ≤ 0.001), and % neutrophils (*p* ≤ 0.001), and significantly lower T-lymphocyte count (*P* = 0.001), CD4+ T lymphocyte count (*P* = 0.002), albumin (*p* ≤ 0.001), sodium (*p* ≤ 0.001), Hb (*P* = 0.002), and Plt count (*P* = 0.019).

In the logistic regression analysis, 11 laboratory variables (PCT, SF, sIL-2R, albumin, direct bilirubin, total bilirubin, sodium, CRP, % neutrophils, Hb, and Plt count) were included. Albumin, % neutrophils, and Hb were found to be independent predictors of IVIG resistance in multivariable logistic regression analysis models (IVIG responders, *n* = 478; IVIG nonresponders, *n* = 30; missing values, *n* = 257; Hosmer–Lemeshow test = 0.815; Table 2).

**Figure 1:**
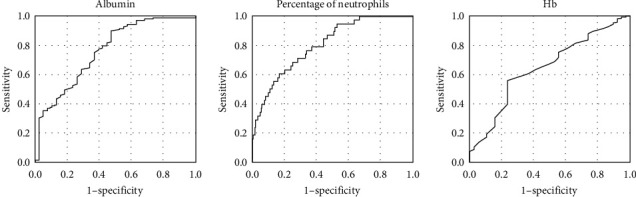
The receiver-operating–characteristics curve for the variables involved in our new risk-scoring system for distinguishing between the responders and the nonresponders.

**Figure 2:**
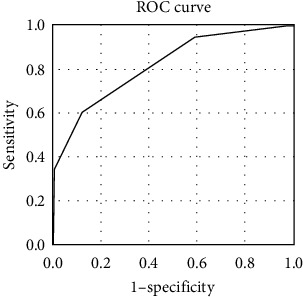
The receiver-operating–characteristics curve for our new risk-scoring system.

Cutoff points for the predictors calculated using the ROC curve, and the sensitivity, specificity, positive predictive value (PPV), and negative predictive value (NPV) are shown in Table 3 and Figure 1. A new risk-scoring system was created as follows based on the odds ratios of the predictors: albumin ≤31.5 g/L (1 point), % neutrophils ≥72.5% (1 point), and Hb ≤105.5 g/L (1 point). The area under the ROC curve (AUC) was 0.818 (95% confidence interval: 0.744–0.892). The new risk-scoring system had a sensitivity of 60.5%, specificity of 87.8%, PPV of 72.2%, and NPV of 96.4%. Based on the Youden values of each cutoff point of this scoring system, participants with scores ≥2 were defined as having a high risk of IVIG resistance (Table 4 and Figure 2).

**Figure 3:**
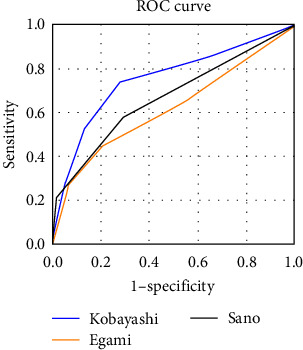
The receiver-operating–characteristics curve for Kobayashi, Egami, and Sano scoring system.

Analysis results of the Kobayashi, Egami, and Sano scoring systems are presented in Table 4 and Figure 3. The Kobayashi scoring system had a sensitivity, specificity, PPV, NPV, and AUC of 26.3%, 94.7%, 22.2%, 95.7%, and 0.752, respectively. The Egami scoring system had a sensitivity, specificity, PPV, NPV, and AUC of 26.3%, 93.1%, 17.9%, 95.7%, and 0.617, respectively. Further, the Sano scoring system had sensitivity, specificity, PPV, NPV, and AUC of 21.1%, 98.2%, 40.0%, 95.6%, and 0.668, respectively.

## 4. Discussion

Accumulating evidence has indicated that IVIG resistance is strongly associated with a high risk of developing CALs [14–16]. Therefore, early identification of IVIG nonresponders is crucial for the providing timely additional treatment. The development of CALs may be affected by hemodynamics after treatment and persistent inflammation. Therefore, the prediction of IVIG resistance is easier than the probability of developing CALs. A risk predictive model whit high sensitivity and specificity to distinguish between IVIG responder and nonresponder may play an important role in guiding clinical therapy. It has been reported that the Kobayashi, Egami, and Sano scoring systems are not suitable for all populations [16–19]; thus, these predictive models to identify patients at risk of IVIG nonresponsiveness may not be applicable to Chinese patients. This was reflected in the low-sensitivity values of these scoring systems observed in our study. Therefore, we aimed to identify a predictive scoring system for IVIG resistance in KD specific to the Chinese population.

In this retrospective study, a new risk-scoring system was developed as follows: albumin ≤31.5 g/L (1 point), % neutrophils ≥72.5% (1 point), Hb ≤105.5 g/L (1 point), and cutoff point ≥2. This system had a higher sensitivity than the Kobayashi, Egami, and Sano scoring systems. Besides, the detection technology of these variables involved in our new risk-scoring system is simple so as to beneficial for use in the vast majority of Chinese hospitals regardless of the level of the hospital. At the same time, the detection cost of these variables is not high, which is beneficial for more patients.

An increasing number of reports have focused on the predictive value of SF in IVIG resistant KD [20–22]. SF plays an important role in the storage of intracellular iron as a cytosolic protein in most tissues. It may be elevated due to factors such as infection, inflammation, and malignancy [23]. In the presence of inflammation, tumor necrosis factor-*α* and interleukin-1*α* released by activated macrophages significantly induce ferritin synthesis [24, 25]. In this retrospective review, significant differences in SF levels were observed between IVIG responders and nonresponders in the Student's *t*-test. However, multivariable logistic regression analysis demonstrated that it was not an independent predictor of IVIG resistance in KD. A possible reason for this may be the small sample size of patients with IVIG resistance in this study. Hence, larger sample sizes should be included in follow-up studies.

The RAISE study in Japan [26] demonstrated that corticosteroid therapy might be beneficial for the primary treatment of severe KD, and that IVIG and prednisolone therapy could significantly decrease the incidence of coronary artery abnormalities. In this study, the differences in laboratory variables (SF, sIL-2R, ALT, total bilirubin, direct bilirubin, CRP, WBC count, % neutrophils, T-lymphocyte count, CD4+ T lymphocyte count, sodium, Hb, and Plt count) were not statistically significant between IVIG nonresponders and the IVIG responders treated with initial IVIG and glucocorticoid therapy (Table S2). This suggests that additional glucocorticoid treatment may reduce the incidence of IVIG resistance in patients with KD. Therefore, the development of a new risk-scoring system to predict IVIG resistance in the Chinese population is important for early recognition and treatment with additional glucocorticoid therapy in patients at increased risk. This may potentially reduce the incidence of CALs.

This study has some limitations. First, because of the early use of glucocorticoids, patients with IVIG resistance accounted for only a small proportion of the total sample size. Second, only patients who received initial treatments for the first 10 days of illness were included, which may have resulted in bias. Therefore, the number of days of illness at the initial treatment was not compared. Third, patients with comorbidities from other febrile illnesses could not be excluded. Considering that the new risk-scoring system was developed in Chinese population, their potential applicability should be further checked in the population from other countries. Despite some limitations, the advantages of this study which was conducted based on the demographic and laboratory variables include the use of organized statistical methods in scoring development, as well as good sensitivity, specificity, and AUC in scoring to predict IVIG resistance.

## 5. Conclusions

This study developed a novel risk-scoring system with higher specificity than the Kobayashi, Egami, and Sano scoring systems, which may help in the early detection of IVIG resistance in Chinese patients with KD. Although this new predictive model requires further validation, it may be useful for improving the prognosis of patients with KD and reducing the risk of associated complications such as CALs.

## Figures and Tables

**Table 1:** Comparison of clinical and laboratory variables between IVIG responders and IVIG nonresponders.

Variables	IVIG responders *n* = 665	IVIG nonresponders *n* = 38	*P* values
Male	403 (60.60%)	26 (68.42%)	0.336
Age (month)	26.54 ± 21.39	32.24 ± 31.47	0.278
ESR (mm/hr)	63.63 ± 26.30	72.26 ± 37.15	0.166
BNP (pg/mL)	159.72 ± 199.13	403.72 ± 840.81	0.091
PCT (ng/mL)	1.13 ± 1.81	2.70 ± 4.44	0.036
SF (ng/mL)	169.84 ± 141.99	294.01 ± 238.23	0.003
T lymphocyte (/*μ*L)	2,147.40 ± 1,153.44	1,446.33 ± 1,064.17	0.001
CD4+ T lymphocyte (/*μ*L)	1,368.83 ± 830.40	912.50 ± 745.04	0.002
SIL-2R (U/mL)	2,187.97 ± 1,435.44	3,293.73 ± 2,469.47	0.021
IL-6 (pg/mL)	39.24 ± 76.48	98.13 ± 162.75	0.058
ALT (U/L)	35.51 ± 49.80	69.63 ± 92.54	0.030
AST (U/L)	38.66 ± 42.57	54.89 ± 67.68	0.152
Albumin (g/L)	37.09 ± 4.40	32.31 ± 5.18	*P* ≤ 0.001
Total bilirubin (*μ*mol/L)	6.39 ± 5.89	13.39 ± 14.16	0.004
Direct bilirubin (*μ*mol/L)	2.83 ± 4.00	8.76 ± 11.98	0.004
Sodium (mmol/L)	135.95 ± 2.5	134.21 ± 3.01	*P* ≤ 0.001
CRP (mg/L)	51.17 ± 44.50	86.37 ± 65.68	0.002
WBC (×10^9^/L)	11.84 ± 5.21	19.89 ± 9.92	*P* ≤ 0.001
Percentage of neutrophils (%)	51.28 ± 19.82	72.96 ± 14.98	*P* ≤ 0.001
Hb (g/L)	106.72 ± 10.81	101.08 ± 10.57	0.002
Plt (×10^9^/L)	385.37 ± 127.59	334.45 ± 164.30	0.019

Data is presented as number (%) or mean ± standard deviation.

**Table 2:** Multivariable logistic regression analysis for prediction of IVIG nonresponsiveness in KD.

	Logistic coefficient (*β*)	SE	*P* values	OR	95% CI	
					Lower	Upper
PCT	0.070	0.069	0.313	1.072	0.936	1.228
SF	0.001	0.001	0.582	1.001	0.998	1.003
SIL-2R	*P* ≤ 0.001	*P* ≤ 0.001	0.938	1.000	1.000	1.000
Albumin	−0.137	0.055	0.012	0.872	0.783	0.971
Direct bilirubin	0.037	0.044	0.393	1.038	0.953	1.130
Total bilirubin	0.011	0.034	0.760	1.011	0.945	1.081
Sodium	−0.056	0.077	0.467	0.945	0.812	1.100
CRP	−0.006	0.005	0.203	0.994	0.984	1.003
Percentage of neutrophils	0.068	0.018	*P* ≤ 0.001	1.070	1.033	1.108
Hb	−0.061	0.029	0.037	0.941	0.889	0.996
Plt	0.001	0.002	0.603	1.001	0.997	1.004

Data is presented as number (%) or mean ± standard deviation.

**Table 3:** ROC curve for variables level for distinguishing between the IVIG responders and the nonresponders.

	Cutoff	Sensitivity (%)	Specificity (%)	PPV (%)	NPV (%)
Albumin (g/L)	31.5	89.9	52.6	21.7	97.1
Percentage of neutrophils (%)	72.5	60.5	83.0	16.5	97.2
Hb (g/L)	105.5	55.8	76.3	9.0	97.6

PPV, positive predictive value; NPV, negative predictive value.

**Table 4:** Sensitivity and specificity of Kobayashi scoring system, Egami scoring system, Sano scoring system, and the new scoring system.

	Cutoff (point)	AUC	Sensitivity (%)	Specificity (%)	PPV (%)	NPV (%)
Kobayashi score	4	0.752	26.3	94.7	22.2	95.7
Egami score	3	0.617	26.3	93.1	17.9	95.7
Sano score	2	0.668	21.1	98.2	40.0	95.6
New score	2	0.818	60.5	87.8	72.2	96.4

PPV, positive predictive value; NPV, negative predictive value.

## Data Availability

The clinical and laboratory data of children with KD used to support the findings of this study are available from the corresponding author (haiguo_yu@njmu.edu.cn; zhidan1728@163.com) upon request.

## Additional Points

*Key Points*. Approximately 10%–20% of patients with KD are nonresponsive to IVIG treatment, placing them at higher risk of developing coronary heart lesions. We constructed a new predictive scoring system with higher specificity for patients with KD in China. We believe that our study makes a significant contribution to the literature because it describes a novel scoring system that may aid in the early detection of nonresponsiveness to IVIG treatment, thus facilitating timely intervention and improving the health outcomes of patients with KD. Further, our findings contribute to the development of pediatric medicine and research, and will help pediatricians in predicting IVIG resistance in KD with greater specificity.

## Supplementary Materials

Supplementary Materials Table S1: commonly used risk-scoring systems for IVIG nonresponsiveness in KD. The three risk-scoring systems including Kobayashi scoring system (AST ≥100 IU/L: 2 points, sodium ≤133 mmol/L: 2 points, days of illness at initial treatment ≤4: 2 points, neutrophils ≥80%: 2 points, CRP ≥100 mg/L: 1 point, Plt ≤300 × 10^9^/L: 1 point, Age ≤12 month: 1 point, cutoff ≥4 points), Egami scoring system (ALT ≥80 IU/L: 2 points, CRP ≥80 mg/L: 1 point, days of illness at initial treatment ≤4: 1 point, Plt ≤300 × 10^9^/L: 1 point, Age ≤6 month: 1 point, cutoff ≥3 points), and Sano scoring system (AST ≥200 IU/L: 1 point, CRP ≥70 mg/L: 1 point, total bilirubin ≥15.39 umol/L: 1 point, cutoff ≥2 points) were listed in Table S1. Table S2: comparison of clinical and laboratory bariables between IVIG nonresponders and the IVIG responders treated with initial IVIG together with glucocorticoid therapy. The differences in laboratory variables (SF, sIL-2R, ALT, total bilirubin, direct bilirubin, CRP, WBC count, % neutrophils, T-lymphocyte count, CD4+ T lymphocyte count, sodium, Hb, and Plt count) were not statistically significant between IVIG nonresponders and the IVIG responders treated with initial IVIG and glucocorticoid therapy.
