# Supplementary material for: Novel Predictive Scoring System for Intravenous Immunoglobulin Resistance Helps Timely Intervention in Kawasaki Disease: The Chinese Experience
Source: J Immunol Res. 2023 Aug 9;2023:6808323. doi: 10.1155/2023/6808323 (PMC10432039; doi:10.1155/2023/6808323)
Supplement: Supplementary Materials — Table S1: commonly used risk-scoring systems for IVIG nonresponsiveness in KD. The three risk-scoring systems including Kobayashi scoring system (AST ≥100 IU/L: 2 points, sodium ≤133 mmol/L: 2 points, days of illness at initial treatment ≤4: 2 points, neutrophils ≥80%: 2 points, CRP ≥100 mg/L: 1 point, Plt ≤300 × 109/L: 1 point, Age ≤12 month: 1 point, cutoff ≥4 points), Egami scoring system (ALT ≥80 IU/L: 2 points, CRP ≥80 mg/L: 1 point, days of illness at initial treatment ≤4: 1 point, Plt ≤300 × 109/L: 1 point, Age ≤6 month: 1 point, cutoff ≥3 points), and Sano scoring system (AST ≥200 IU/L: 1 point, CRP ≥70 mg/L: 1 point, total bilirubin ≥15.39 umol/L: 1 point, cutoff ≥2 points) were listed in Table S1. Table S2: comparison of clinical and laboratory bariables between IVIG nonresponders and the IVIG responders treated with initial IVIG together with glucocorticoid therapy. The differences in laboratory variables (SF, sIL-2R, ALT, total bilirubin, direct bilirubin, CRP, WBC count, % neutrophils, T-lymphocyte count, CD4+ T lymphocyte count, sodium, Hb, and Plt count) were not statistically significant between IVIG nonresponders and the IVIG responders treated with initial IVIG and glucocorticoid therapy. [file 6808323.f1.docx]

**Supplementary Table 1. Commonly Used Risk Scoring Systems for IVIG Non-responsiveness in KD**

| Kobayashi score  (Cut-off ≥4 points) | | Egami score  (Cut-off ≥3 points) | | Sano score  (Cut-off ≥2 points) | |
| --- | --- | --- | --- | --- | --- |
| Variables | Points | Variables | Points | Variables | Points |
| AST ≥100 IU/L | 2 | ALT ≥80 IU/L | 2 | AST ≥200 IU/L | 1 |
| Sodium ≤133 mmol/L | 2 | CRP ≥80 mg/L | 1 | CRP ≥70 mg/L | 1 |
| Days of illness at initial treatment ≤4 | 2 | Days of illness at initial treatment ≤4 | 1 | Total bilirubin ≥15.39 umol/L | 1 |
| Neutrophils ≥80 % | 2 | Plt ≤300*10^9/L | 1 |  |  |
| CRP ≥100 mg/L | 1 | Age ≤6 month | 1 |  |  |
| Plt ≤300*10^9/L | 1 |  |  |  |  |
| Age ≤12 month | 1 |  |  |  |  |

**Supplementary Table 2. Comparison of Clinical and Laboratory Variables Between IVIG Non-responders and the IVIG Responders Treated with initial IVIG together with glucocorticoid therapy.**

| Variables | IVIG non-responders  IVIG | IVIG Responders  IVIG + glucocorticoid | *P* values |
| --- | --- | --- | --- |
| Male | 26(68.42 %) | 33(55.93%) | 0.219 |
| Age (month) | 32.24±31.47 | 30.73±26.39 | 0.800 |
| PCT (ng/mL) | 2.70±4.44 | 7.82±16.78 | 0.029 |
| SF (ng/mL) | 294.01±238.23 | 268.70±199.96 | 0.582 |
| T lymphocyte (/ul) | 1446.33±1064.17 | 1418.79±1633.55 | 0.931 |
| CD4+T lymphocyte (/ul) | 912.50±745.04 | 883.90±1065.98 | 0.893 |
| SIL-2R (U/mL) | 3293.73±2469.47 | 3478.14±2316.23 | 0.754 |
| ALT (U/L) | 69.63±92.54 | 78.76±102.91 | 0.658 |
| Albumin (g/L) | 32.31±5.18 | 35.06±5.82 | 0.020 |
| Total bilirubin (umol/L) | 13.39±14.16 | 16.13±19.29 | 0.453 |
| Direct bilirubin (umol/L) | 8.76±11.98 | 10.87±17.63 | 0.520 |
| Sodium (mmol/L) | 134.21±3.01 | 134.43±3.60 | 0.753 |
| CRP (mg/L) | 86.37±65.68 | 84.42±59.00 | 0.880 |
| WBC (*10^9/L) | 19.89±9.92 | 17.22±7.37 | 0.160 |
| % Neutrophils (%) | 72.96±14.98 | 73.36±15.82 | 0.902 |
| Hb (g/L) | 101.08±10.57 | 104.19±11.15 | 0.175 |
| Plt (*10^9/L) | 334.45±164.30 | 354.76±154.19 | 0.538 |

**Data is presented as number (%) or mean ± standard deviation.**
